# The Axial Frontonasal Flap for Nasal Tip Defect: A Single Centre Experience

**DOI:** 10.7759/cureus.5892

**Published:** 2019-10-11

**Authors:** Badi F Aldosari

**Affiliations:** 1 Otolaryngology, King Saud University, Riyadh, SAU

**Keywords:** nose, reconstruction, flap, frontonasal, nasal tip defect

## Abstract

Objectives

Skin defects of the nose can be a surgical challenge for facial plastic surgeons. Often, invasive surgery of the nose is accompanied by aesthetic issues for the patient. Hence, following invasive surgery of the nose, a reconstructive procedure should be performed to correct the morphological and aesthetic issues. This can help to restore the aesthetic features of the nose. Of the several reconstructive procedures available for nasal tip defects, the axial frontonasal flap is regarded as a good option for nasal tip reconstruction. Herein, we describe our experience with the axial frontonasal flap for the reconstruction of nasal tip defect in 15 patients. We also would specify the indications for the use of this flap and describe in detail the surgical technique.

Materials and Methods

This was a retrospective study of 15 patients who underwent reconstruction of nasal tip defect with an axial frontonasal flap between 2012 and 2015. In all the patients, the defects were located on the nasal tip. The measurement of the nasal tip defect in all the cases was ≥ 1.4 cm in their shortest diameter. The patients were followed up to 12 months after surgery.

Results

The axial frontonasal flaps for all the patients survived completely without any complications. In all patients, the follow-up surgical result was rated as good or excellent by the patients and surgeons.

Conclusions

Based on the results of our review of cases, we highly recommend the axial frontonasal flap as an option for the reconstruction of nasal tip defects measuring ≥ 1.4 cm in diameter.

## Introduction

Reconstruction of skin defects of the nasal tip can be a challenging task for facial plastic surgeons, as the nasal tip is a prominent facial landmark. Furthermore, as the irregularities in skin color, texture, thickness, and contour are very visible, the reconstruction could be more difficult. Hence, invasive surgery of the nose should be followed by a reconstructive procedure that should attempt to correct the morphological and aesthetic features as much as possible [1].

Aesthetic units are crucial when reconstructing nasal tip defects. It is essential to obtain skin samples that match the local tissues with respect to color and texture. For the reconstruction, the adjacent skin tissue is preferred, as it bears the same color and texture as the nasal tip [2].

Over the years, numerous flaps for the reconstruction of nasal tip defects have been described. These flaps are all based on the mobilization of the skin of the dorsum and the glabellar region [2]. A common complication encountered in different flaps is the rotation of the local flap that may result in ‘dog-ear’ or ‘trap-door’ deformities. Also, most flaps cannot cover large nasal tip defects. These issues can be overcome using the axial frontonasal flap [1].

The axial frontonasal flap is a local rotation flap that uses nasal skin from around the axial defect (i.e., the remaining nasal tip, the dorsum, and the glabellar area) to reconstruct the nasal tip defect. The procedure could be completed in a single stage. The first axial frontonasal flap was proposed by Marchac in 1970 [3-4], and in 1993, a revised Marchac flap was suggested by de Fontaine et al. [5].

In this study, we describe our experience with the use of an axial frontonasal flap for the reconstruction of a nasal tip defect. We would also specify the indications for this flap and describe in detail the surgical technique.

## Materials and methods

A total of 15 patients with nasal tip defect measuring ≥ 1.4 cm in their shortest diameter were included in this study. These patients underwent reconstruction of nasal tip defect with axial frontonasal flap at the Division of Otorhinolaryngology-Head and Neck Surgery, University of Montreal, Montreal, Québec, Canada. Four patients were women and 10 were men, aged between 65 and 94 years with a mean age of 75 years. To be included in the study, the patients must have had a nasal tip defect of 1.4 cm or more in their shortest diameter (the smallest diameter 1.4 cm and the largest diameter 2.5 cm) (Table 1). Following surgery, all the flaps survived completely. Also, we noticed no skin necrosis, postoperative hematomas, or infection. Moreover, there was no distortion of the nasal tip or alar rim. The postoperative course of the patients was generally rapid and well-tolerated. In all of the patients, the surgical result was rated as good or excellent by the patients and surgeons.

**Table 1:** Demographics, Defect Size, and Flap Design

Age	Sex	Location	Size	Anesthesia	Flap design	Complications postop
94 y	M	Nasal tip	1.9 cm x 1.4 cm	Local	de Fontaine	No
82 y	F	Nasal tip	1.8 cm x 1.4 cm	Local	de Fontaine	No
83 y	F	Nasal tip	2 cm x 1.5 cm	Local	de Fontaine	No
88 y	M	Nasal tip	2.5 cm x 1.6 cm	Local	de Fontaine	No
65 y	M	Nasal tip	1.7 cm x 1.6 cm	Local	de Fontaine	No
83 y	M	Nasal tip	1.8 cm x 1.5 cm	Local	de Fontaine	No
74 y	M	Nasal tip	2.5 cm x 2.4 cm	Local	de Fontaine	No
79 y	M	Nasal tip	1.8 cm x 1.7 cm	Local	de Fontaine	No
84 y	M	Nasal tip	1.8 cm x 2.2 cm	Local	de Fontaine	No
75 y	F	Nasal tip	2.1 cm x 2 cm	Local	de Fontaine	No
79 y	M	Nasal tip	2.3 cm x 2.4 cm	Local	de Fontaine	No
80 y	M	Nasal tip	1.7 cm x 2 cm	Local	de Fontaine	No
77 y	F	Nasal tip	1.5 cm x 2.4 cm	Local	de Fontaine	No
86 y	M	Nasal tip	2.2 cm x 2.4 cm	Local	de Fontaine	No
90 y	F	Nasal tip	2.3 cm x 2.5 cm	Local	de Fontaine	No

## Results

The axial frontonasal flap is a local rotation flap that uses nasal skin from the vicinity of the defect (the remaining tip, the dorsum, and the glabellar area) in order to reconstruct a tip defect. The procedure is usually completed in a single stage. The operation is performed under local anesthesia.

In our series of cases, the flap was a pivotal flap. The pedicle of the flap was always located on the same side of the defect. A curvilinear incision was drawn from the inferior portion of the defect laterally until it met the junction of two aesthetic subunits (the tip and the ala) and then to the junction of the cheek and the nose. From this point, the incision was directed upward toward the superior aspect of the glabella, keeping 0.5 cm distance medially while passing the medial canthus. The flap length decreased progressively as the flap rotated about its pivotal point. Hence, the length of the flap must be sufficient to compensate for this shortening. The supplemental flap height was gained from the glabellar extension by creating an incision toward the contralateral medial canthus, creating a 30° to 45° angle [6].

**Figure 1:**
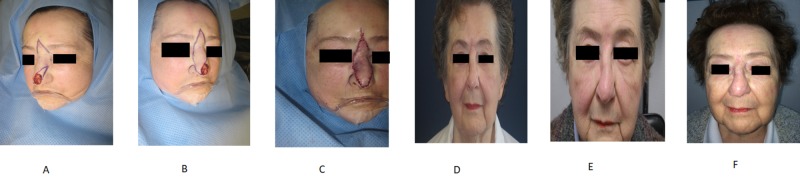
A 82-year-old woman with a basal cell carcinoma of the tip of the nose A-C) Intraoperative views; D) three months after surgery; E) six months after surgery; F) one year after surgery

The flap was elevated in the subcutaneous plane and beneath the musculature of the nose in the glabellar part and nasal part, respectively. Complete mobilization of the pedicle was necessary for proper tissue movement. The dog-ear was always resected, removing a triangle of skin towards the medial canthus on the side of the defect. Deep sutures were necessary to position the flap. Skin incisions were closed with 5-0 plain interrupted sutures. The donor site in the glabella may be closed by V-Y advancement (Figure 1A-F) [6].

## Discussion

Skin defects of the nasal tip can be repaired in a number of ways - with full-thickness skin grafts, local flaps (such as a bilobed flap), or regional flaps (such as a forehead flap). These methods depend on the size of the defect. Local flaps present many advantages; they are one-stage procedures that use adjacent skin which is often similar in color and texture. A wide range of local flaps has been described to cover nasal tip defects, from single and bilobed rotation flaps to forehead flaps, V-Y advancement flaps, etc. The forehead flap is the preferred reconstruction procedure, especially in the case of extensive nasal soft tissue loss. Other local flaps all cause a certain degree of distortion of the nasal profile, and they can create scars that lie within the nose aesthetic subunits, making them quite visible. Many local flaps do not respect the subunit principle. The original design of the axial frontonasal flap by Marchac also creates a scar crossing the dorsum of the nose [4]. The scar in the frontonasal flaps is negligible and well-tolerated if the flap is designed correctly [7]. Soft-tissue triangle defects can be reconstructed with an axial frontonasal flap [7-8]. The bilobed flap is a good choice for repair of a nasal tip defect less than 1.5 cm; if the nasal tip defect is more than 1.5 cm, the forehead flap is an option for reconstruction but the forehead flap is a two-stage procedure. In elderly patients where skin laxity is present and where a local one-stage procedure is preferred, the axial frontonasal flap is a good option and must be included in the reconstruction algorithm.

## Conclusions

Our review of cases has demonstrated that an axial frontonasal flap is an excellent option for single-stage reconstruction of nasal tip defects that measure more than 1.4 cm in diameter in elderly patients with multiple comorbidities. Moreover, the scar was negligible and there was no distortion of nasal tip or alar rim.
